# Immunogenicity of a bivalent protein as a vaccine against *Edwardsiella anguillarum* and *Vibrio vulnificus* in Japanese eel (*Anguilla japonica*)

**DOI:** 10.1002/mbo3.766

**Published:** 2018-11-16

**Authors:** Songlin Guo, Linlin Hu, Jianjun Feng, Peng Lin, Le He, Qingpi Yan

**Affiliations:** ^1^ Fishery College of Jimei University/Engineering Research Center of Modern Eel Industrial Technology of the Ministry of Education, PRC Xiamen China; ^2^ Jimei University Fujian, Xiamen China

**Keywords:** *Anguilla japonica*, *Edwardsiella anguillarum*, expression, outer membrane protein, vaccine, *Vibrio vulnificus*

## Abstract

The OMPs A (OmpA)—of *Edwardsiella anguillarum* and OmpU of *V. vulnificus* have been proven to be good antigens. In this study, after construction of a vector, a new recombinant Omp (rOMP) containing both OmpA and OmpU was expressed and purified. Then, the Japanese eels (*Anguilla japonica*) were intraperitoneally (i.p.) injected with the phosphate‐buffered saline (PBS group), formalin‐killed‐cell (FKC group) or the recombinant Omp (rOMP group). The stimulation index of the whole blood cells in eels from FKC group was significantly higher than the eels from PBS and rOMP groups at 28 dpi; serum titers of anti‐*E. anguillarum* and anti‐*V. vulnificus* antibody of eels from FKC and rOMP group increased significantly at 21 and 28 dpi; in the rOMP group, eels serum titer stayed at a high level on 42 dpi. The activities of lysozyme in skin mucus, liver, kidney, and serum in three groups exhibited considerable changes. The relative percent survival (RPS) rate of eels from rOMP group were 100% and 83% when challenged with *V. vulnificus* or* E. anguillarum*. These results indicated that inoculation of rOMP would protect Japanese eels against the infection by *E. anguillarum* and *V. vulnificus*.

## 1 INTRODUCTION

Japanese eel (*Anguilla japonica*), a freshwater fish with high economic value, is widely cultured in large areas in China in recent 20 years. The occurrence of bacterial diseases is the common factor that affects Japanese eel production in China. *Edwardsiella anguillarum*, identified as *Edwardsiella tarda* before 2015 (Buján et al., 2018; Shao et al., 2015) and *Vibrio vulnificus* (Esteve‐Gassent, Nielsen, & Amaro, 2003; Haenen et al., 2014; Reichley et al., 2017) are two important bacterial pathogens in eels farming, and cause serious disease and high levels of mortality in cultured eels resulting in considerable economic losses in Chinese aquaculture (Guo, 2006; Guo et al., 2013; 2015). These two pathogens are also recognized as causing serious diseases in a variety of fish species globally (Griffin et al., 2013; Jheng et al., 2015; Lee et al., 2014; Shao et al., 2015; Tsao et al., 2013).

At present, there are limited therapeutants available for use in aquaculture in China, which poses challenges to the prevention and treatment of aquaculture diseases. Vaccination is an important tool in the prevention of infection diseases in aquaculture. Several studies have described the effectiveness of vaccines in eels and other fisheries, such as formalin‐killed‐cells (FKC) (Bastardo, Ravelo, Castro, Calheiros, & Romalde, 2012; Esteve‐Gassent, Fouz, & Amaro, 2004; Shoemaker, LaFrentz, & Klesius, 2011), live attenuated vaccine (Guo et al., 2014; Loessner et al., 2008), and subunit vaccines (Tang, Zhan, Sheng, & Chi, 2010; Tian, Xu, Lin, Gong, & Lin, 2010) for the control of *Edwardsiella* and *Vibrio* species. Outer membrane proteins (OMPs) are considered to be good vaccine candidates for the protection against infection (Cheng, Chu, Wang, Peng, & Li, 2018; Guan, Xiong, Huang, & Guo, 2011; Kawai, Liu, Ohnishi, & Oshima, 2004; Li et al., 2013; Tang et al., 2010; Tian et al., 2010). Studies on the immunogenicity of the two proteins, OmpA from *E. anguillarum* and OmpU from *V. vulnificus* had been previously evaluated in the Japanese eel (Duan, 2016; Duan et al., 2018; He, Duan, Feng, Peng, & SongLin, 2018). Experimental vaccines based on these antigens were demonstrated to have good immunogenicity after the vaccinated Japanese eels were challenged by *E. anguillarum* or *V. vulnificus*, but the immune effects of a fusion OMP from the two pathogens had not been described. The advantage of a combination of the two OMPs would be to protect animals against two pathogens, as well as to reduce the workload of expression and purification of multiple proteins (Guo et al., 2013; 2015).

The immunogenicity of bivalent OmpII‐OmpS2 (OmpII from *Aeromonas hydrophila* and OmpS2 from *E. anguillarum*) and OmpII‐OmpU (OmpII from *A. hydrophila* and OmpU from *V. vulnificus*) were firstly evaluated (Guo et al., 2013; 2015). The result showed immunogenicity of OmpS2 was relatively weak since the relative protection rate (RPS) against *E. anguillarum* challenge was only 37.5%. Compare with two previous studies, OmpA instead of OmpS2 of *E. anguillarum* was used, and fish used in this study was local eels (Japanese eel) instead of exotic eels (American eel). Freund's incomplete adjuvant was added only in the OMP group in two previous studies, which could not remove differences of immune protection caused by adjuvant.

In order to develop a new recombinant OMP (rOMP) vaccine to protect Japanese eels from infection of *E. anguillarum* and *V. vulnificus,* we vaccinated eels with an expressed fusion of two OMPs that includes both OmpA (*E. anguillarum*) and OmpU (*V. vulnificus*). The immunogenicity of our vaccine preparation and its effectiveness with respect to protection of Japanese eels against ip challenge with these two bacteria was demonstrated via live bacterial challenge post vaccination. This work is unique and advances the field of aquaculture vaccination as a means of protection against economically damaging common pathogens.

## 2 MATERIALS AND METHODS

### 2.1 Fish, bacteria strains, and plasmid

Healthy Japanese eels (*A. japonica*) with a mean body mass of 50 g were obtained from Fuqin, China. The fish were randomly assigned into three groups of 60 fish and maintained in 1,000‐L tanks. Fish were allowed to adapt to experimental conditions (aerated, 26°C) for 7 days before immunization. Fish were fed with pellets (Tianma Feed Co., Ltd. in China) daily, fish feces and feed remnants were removed within 3 hr of feeding.

Pathogenic of *E. anguillarum* B79 isolated from the kidney of infected eels with ulcers (Guo et al., 2013) and *V. vulnificus* B88 isolated from the infected liver (Guo et al., 2015) were used in this study. The bacterial strains were stored at −70°C in saline containing 20% glycerol. The TA Cloning Vector pMD™19‐T (Simple) was obtained from TaKaRa Biotechnology Dalian Co., Ltd. (China), and the reformed expression vector pGex‐2T‐His was obtained from “Third Ocean Research Institute of the State Oceanic Administration” and adding six‐His tag to the C terminal end of the plasmid, and the restriction endonuclease of *BamH*I was substitute by *Sal*I.

### 2.2 Preparation of FKC of *E. anguillarum* and *V. vulnificus*

*Edwardsiella anguillarum* and *V. vulnificus* were incubated in Tryptone soya broth (TSB) at 28°C for 24 hr. The bacterial cells were harvested by centrifugation at 5,000× g for 10 min. After washing 3 times with PBS (10 mmol/L, pH 7.4, NaCl 8 g; KCl 0.2 g; Na_2_HPO_4_•12H_2_O 3.63 g; KH_2_PO_4_ 0.24 g; H_2_O 1,000 ml), the bacterial cells were resuspended in PBS to a concentration of 1.0 × 10^9^ cfu/ml. Cells were inactivated by the addition of formaldehyde 0.4% (v/v), followed by incubation at 28°C for 24 hr. The inactivation of FKC was confirmed by plating method.

### 2.3 Construction of the expressed vector of pGex‐2T‐OmpU‐OmpA‐His

DNA was extracted from fresh overnight culture of *V. vulnificus* and *E. anguillarum* using a conventional phenol‐chloroform DNA extraction method (Mata et al., 2004). DNA purity was confirmed by agarose gel electrophoresis. The extracted DNA was used as a template to amplify the *OmpU* and *OmpA* from *V. vulnificus* and *E. anguillarum,* respectively. The forward primers used to amplify *OmpU* and *OmpA* gene were F1 (5′ACT AAC CCA TCA TGG AAC TTT GG3′) and F2 (5′ ACA CCT ATC ATT AGG GCG TGC3′), respectively, and the reverse primers were R1 (5′AGC TCG ATG TGA AAA GTG AAG CG 3′) and R2 (5′ GAA CTC GGC GTG AGA CAG A 3′), respectively. The amplification protocol for *OmpU* and *OmpA* were referenced as Guo et al (2015).

Primers to amplify a 657‐bp DNA fragment of *OmpU* of *V. vulnificus* (307–963 bp, GenBank accession number: KY072957, 1,023 bp) were F3 (5′ CCG GAA TTC TAC GCA GGT CTA GGC GGC AAG T 3′, adding a 6‐bp restriction site for *Eco*RI and a 3‐bp protective base at the 5′ end) and R3 (5′ACC CGA GCC ACC ACC GCC CGA GCC TAT ACG AGC GTA GCC AGC ACC GCC AAC T 3′, adding a 21‐bp DNA adapter and a 9‐bp protective base at the 5′ end). Primers for amplification of a 687 bp fragment of *OmpA* from *E. anguillarum* (364–1,050 bp, GenBank accession number: KY072958, 1,056 bp) were F4 (5′ TCG GGC GGT GGC GGC TCG GGT GGC GGA TCA GTA TGG CGT TCT GAT ATC CAC GG 3′, adding a 21‐bp DNA adapter and 9‐bp protective base at the 5′ end) and R4 (5′ CGC GTC GAC CTG CGG CTG AGA AAC TTC TTC TT, adding a 6‐bp restriction site for *Sal*I and a 3‐bp protective base at the 5′ end).

The 696 bp (657 + 21+9 + 6+3 = 696) *OmpU* fragment from *V. vulnificus* was ligated with the 726 bp (687 + 21+9 + 6+3 = 726) *OmpA* fragment from *E. anguillarum*, and cloned into the pMD19‐T vector, both the pMD19‐T vector (containing the ligated 1,401 bp [696 + 726–21 = 1,401] OmpU‐OmpA DNA sequence) and the plasmid vector pGex‐2T‐His were simultaneously digested by *Eco*RI and *Sal*I. The fusion *OmpA*‐*OmpU* DNA fragment and the linearized expression plasmid were gel purified with a gel extract kit (Qiagen) and subsequently ligated with T4 DNA ligase (a molar ratio of 4:1) at 4°C for 12 hr. The sequence of the pGex‐2T‐*OmpU‐OmpA*‐His plasmid was confirmed through enzyme digestion, colony PCR, and subsequent DNA sequencing (Invitrogen).

### 2.4 Expression, purification, and theoretical immunogenicity analysis

The summary of the preparation of the rOMP was given as follows: the pGex‐2T‐*OmpU‐OmpA*‐His plasmid was transformed into *E. coli* BL21 for expression (DE3, Novagen), as described by Guo et al (2015). The rOMP was purified using Ni^2+^‐NTA resin, followed by renaturation and lyophilization. SDS‐PAGE gel electrophoresis was used to determine the purity and the molecular mass of the lyophilized protein. Prior to SDS‐PAGE analysis, the lyophilized rOMP was resuspended in PBS (10 mmol/L, pH 7.4) in a final concentration of 1 mg/ml rOMP. The theoretical antigen epitops of the rOMP were predicted by Jameson‐Wolf method (1988) of the Protean program using DNAstar software (Ahmad, Eweida, & Sheweita, 2016).

### 2.5 Vaccination and sampling

One mg/ml rOMP, 1.0 × 10^9^ cfu/ml FKC (50/50 mix of the two bacterial strains) and sterile PBS were mixed with the same volume of Freund's incomplete adjuvant, respectively. Fish in each treatment group were immunized by intraperitoneal (i.p.) injection with 0.2 ml recombinant protein (OMP group), 5.0 × 10^8^ cfu/ml FKC (FKC Group), and PBS (Control group). At 14, 21, 28, and 42 days post immunization (dpi), five eels from each group were sampled at random and euthanized with a lethal dose of Eugenol (10 ppm). Samples of blood, liver, kidney, and skin mucus were obtained from each fish by aseptic techniques (Esteve‐Gassent et al., 2003). Each blood sample was collected in two centrifuge tubes. One tube containing 10 µl heparin (1 iu/µl)was used to collect blood cells; another tube was used to collect serum for determination of antibody titers and lysozyme activity (Guo et al., 2014). Samples of skin mucus, kidney, and liver tissue (100 mg/sample) were homogenized in 0.5 ml of PBS (10 mmol/L, pH 7.4), centrifuged at 10,000 g for 15 min. The final supernatant was collected for determination of lysozyme activity.

### 2.6 Bacterial challenge and determination of relative percent survival (RPS)

Twenty fish from each group were intraperitoneal injection with 0.2 ml of either 2.0 × 10^6^ cfu/ml of *E. anguillarum* (10 fish) or 6.0 × 10^8^ cfu/ml of *V. vulnificus* (10 fish) on 28 dpi and then maintained in six tanks at 26°C. The cumulative mortality of the eels (%) was recorded daily for 14 days. The RPS rate of groups OMP and FKC was calculated as: RPS = (1 − mortality of vaccinated group ÷ mortality of control group) × 100% (Amend, 1981).

### 2.7 Proliferation assay of whole blood cells

The WBC suspension was prepared as previously described (Guo et al., 2015). For each sample, 200 µl of WBC suspension was distributed into 4 wells (50 µl/well), two of these wells received 50 µl of culture solution A (containing 100 µg/ml ConA) and the other two received 50 µl of culture solution B (added no ConA). After incubating at 25°C for 66 hr, 15 µl of MTT (5 mg/ml, dissolving 0.1 g of 3‐(4, 5‐dimethylthiazol‐2‐yl) ‐2, 5‐diphenyltetrazolium bromide [MTT, Biomal] in 20 ml of 10‐mmol/L PBS [pH = 7.4]) was added to each well, and then incubated at 25°C for 4 hr, centrifuged at 2,500 g for 15 min and discarded the supernatant. To each well, 200 µl of DMSO (dimethylsulfoxide, Sigma) was then added with sufficient mixing. The plate was read with a microplate reader (Synergy™ HT, BIO‐TEK) at 570 nm. The value of the stimulation index (SI) of each sample was calculated as: SI = (optical density of one sample with ConA ÷ optical density of one sample without ConA) − 1 (Guo et al., 2015).

### 2.8 Determination of antibody titer

The antibody titer against different antigens of five fish collected from each group at 14, 21, 28, and 42 dpi were assayed by ELISA. In short, 96‐well plates were coated with either purified recombinant proteins (OmpA and OmpU) for the OMP group or a boiled suspension of *E. anguillarum* and *V. vulnificus* (1.0 × 10^8^ cfu/ml) for the FKC and PBS groups (Mao, Yu, You, Wei, & Liu, 2007). Twofold serial dilutions of the sera were added to duplicate wells of the plates with the first dilution of 1:16. Antibodies binding to the antigen were detected using rabbit‐anti‐eel polyclonal antibody (1:400 dilution), followed by goat‐anti‐rabbit IgG conjugated with horse‐radish peroxidase (HRP, Thermo, 1:2000 dilution). Color development was facilitated by the addition of O‐phenylenediamine (OPD, Sigma) for 10 min and followed by deactivation with 2.0 mol/L H_2_SO_4_. Plates were read on a microplate reader (Synergy™ HT) at 492 nm. The Log2 was used to convert the value of the serum titer. Five fish were bled prior to immunization to obtain sera for use as a negative control.

### 2.9 Determination of the lysozyme activity

The lysozyme activities in the liver, skin mucus, serum, and kidney were examined following the manufacture's recommended procedure for the Lysozyme Activity Kit (Nanjin Jianchen Ltd.; Guo et al., 2013).

### 2.10 Statistical analysis

Data are shown as the means ± *SE* Statistical analysis was conducted using SPSS 13.0 software for windows 7. The significance (*p* < 0.01 or <0.05) is measured by ANOVA (Duncan method). The RPS between samples of different groups at each time point post immunization were analyzed using *χ* ^2^ test (chi‐square).

## 3 RESULTS

### 3.1 Construction of the expressed vector

**Figure 1:**
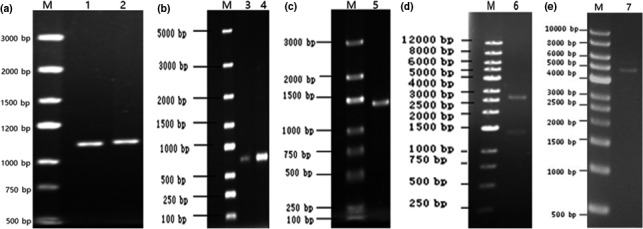
Amplified and combined of partial sequences of *OmpU* and *OmpA*. (a) The full‐length gene sequences of *OmpU* (lane 1) and *OmpA* (lane 2). (b) The partial gene sequences of 696 bp *OmpU* (lane 3) and 726 bp *OmpA* (lane 4). (c) The ligation fragment (lane 5) of *OmpU‐OmpA*. (d) The digestion fragment (digested by *Eco*R I and *Sal* I) of the ligated *OmpU‐OmpA* gene after it cloned into pMD19‐T vector (lane 6). (e) The digested pGex‐2T‐His vector (lane 7). M: DNA Marker

The full‐length gene sequences of *OmpU* and *OmpA* were successfully amplified from the genomic DNA of *V. vulnificus* B88 and *E. anguillarum* B79 (Figure 1a, lane 1 and 2). The partial gene sequences of *OmpU* (696 bp) and *OmpA* (726 bp) were successfully amplified from the purified PCR products of the full‐length gene sequences (Figure 1b, lane 3 and 4). The two partial sequences were ligated through the DNA adapter (Figure 1c, lane 5) and cloned onto the pMD19‐T vector. The ligated *OmpU‐OmpA* gene was cut from the pMD19‐T vector by EcoR I and Sal I (Figure 1d, lane 6), and cloned into the pGex‐2T‐His vector (Figure 1e, lane 7), resulting in the new recombinant pGex‐2T‐*OmpU‐OmpA*‐His plasmid. The restriction enzyme digest, colony PCR assay, and DNA sequencing results showed that the expression plasmid pGex‐2T‐*OmpU‐OmpA*‐His was successfully constructed.

### 3.2 Expression, purification, and immunogenicity analysis of the rOMP

**Figure 2:**
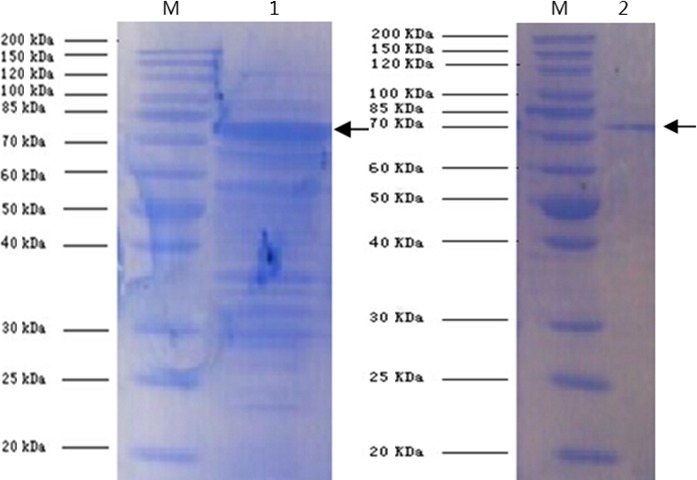
SDS‐PAGE electrophoresis analysis of the expressed and purified expression OMP. M: protein Marker, 1: The expressed rOMP (arrow) after lyses whole cells of the expressed *E. coli* BL21; 2: the rOMP (arrow) purified by Ni2‐NTA method

**Figure 3:**
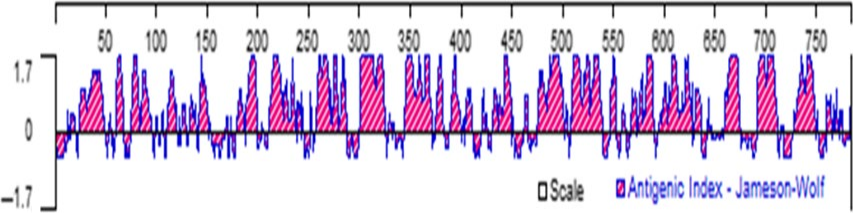
Immunogenicity prediction of the rOMP

The expression plasmid pGex‐2T‐OmpU‐OmpA‐His was transformed into *E. coli* (BL21, *DE3*), and the recombinant protein of OmpU‐OmpA was successfully expressed. The lysis of the expressed cells of *E. coli* resulted in the acquisition of rOMP (Figure 2: lane 1). Through the process of balance, adsorption, and elution, the rOMP was purified using Ni^2+^‐NTA agarose resin. After a sonication process of the expressed cells, this purified rOMP was renatured and then lyophilized. Purification of the lyophilized protein was ascertained using the SDS‐PAGE gel electrophoresis (Figure 2: lane 2), and the molecular mass of the rOMP was determined as 77.6 kD. Immunogenicity prediction of the rOMP by the Jomeson‐Wolf method showed that approximately 98% of the antigen was greater than the critical value of 0, indicating that the recombinant protein has good immunogenicity. According to its main peaks, it could be inferred that the protein had at least 15 strong epitopes (Figure 3).

### 3.3 RPS rate

**Figure 4:**
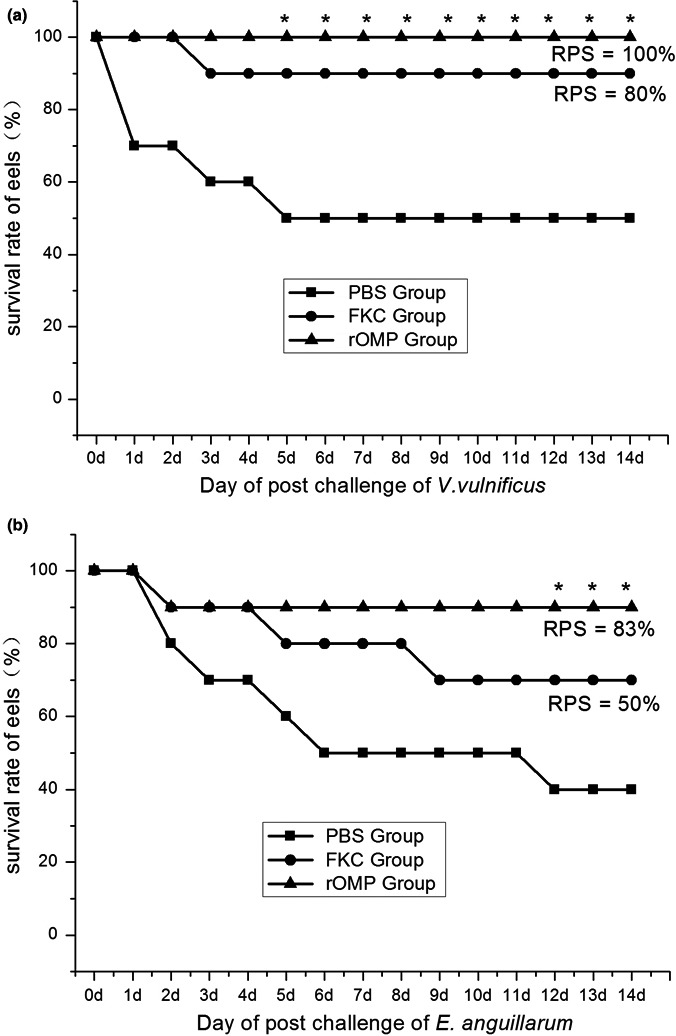
The survival and Relative Percent Survival (RPS) rate of Japanese eels (*A. japonica*) challenged (*i.p*.) by *V. vulnificus* (a) and *E. anguillarum* (b) 28 days post vaccinating, respectively. *: significant difference at *p* < 0.05 between rOMP and PBS control group at one time point post immunization

After infection by *V. vulnificus* on 28 dpi, the mortality rate in the OMP, FKC, and PBS groups was 0%, 10%, and 50%, respectively, and the RPS rate of the OMP and FKC groups, in comparison to the PBS group, were 100% and 80%. Based on the results of a chi‐square test, the survival rate in the OMP group was found to be significantly higher than that in the PBS group (*p* < 0.05) in the period between 5 and 14 dpc (Figure 4a). The infection of counterpart *A. japonica* with *E. anguillarum* showed a similar result, the mortality of 10%, 30%, and 60% were recorded for OMP, FKC, and PBS groups, respectively. The corresponding RPS of the OMP and FKC groups infected by *E. anguillarum* were 83% and 50%, which were lower than those of *V. vulnificus* infection. The survival rate in the OMP group was significantly higher (*p* < 0.05) than that of the PBS group from 12 to 14 dpc (Figure 4b).

### 3.4 Proliferation of the whole blood cells

**Figure 5:**
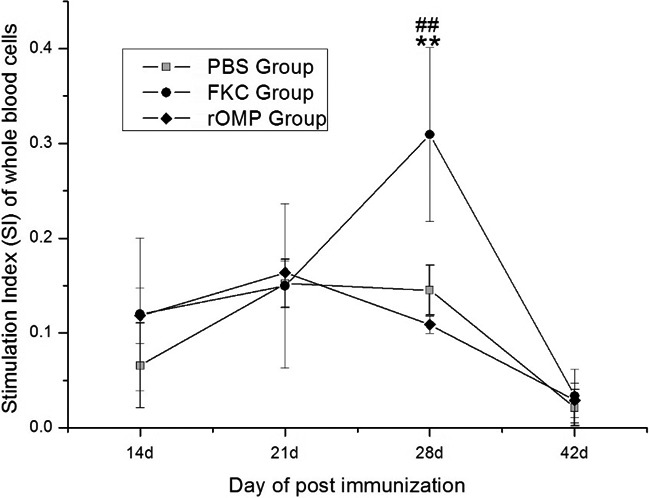
Stimulation index of whole blood cells at different days after eels were immunized with inactivated bacteria vaccine and expressed OMP, data (the mean of SI) are presented as means ± *SE* (*n* = 5). Significant differences were found between groups of immunized fish and group of PBS injected fish at **p < 0.01 and between two immunized groups at ^##^ *p* < 0.01

At 14 dpi, proliferation of whole blood cells in the two test groups was a little higher (*p* > 0.05) than that in the PBS control group. The average SI in WBC of eels in three groups kept at the same level at 21 and 42 dpi although the SI was lower at 42 dpi. Significant differences (**p < 0.01) were found between fish in the PBS and FKC groups and between the two treatment groups (^##^ *p* < 0.01) at 28 dpi (Figure 5).

### 3.5 Antibody titers in response to the immunization with rOMP

**Figure 6:**
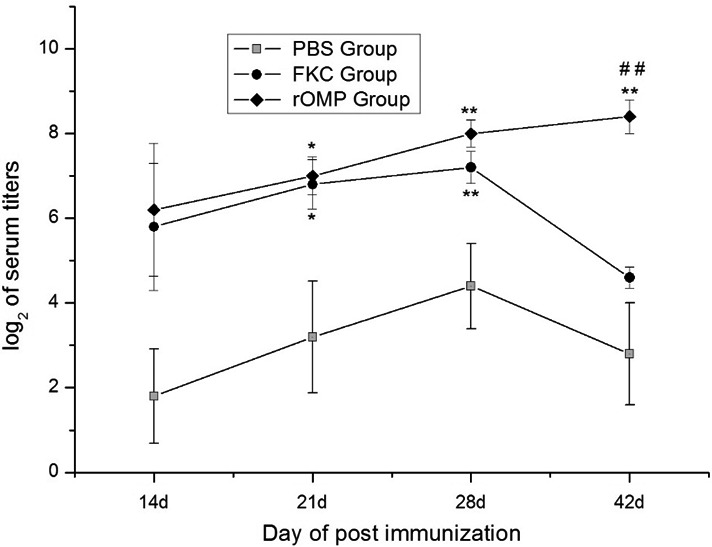
Log2 of serum antigen‐specific antibody responses in immunized eels by ELISA. Data (the mean of OD_492 nm_ readings) are presented as means ± *SE* (*n* = 5). Significant differences were found between groups of immunized fish and group of PBS injected fish at **p < 0.01 and *p < 0.05, and between two treating groups at ^##^ *p* < 0.01

When compared to the control group, the immunized groups showed higher antibody responses (**p < 0.01 or *p* < 0.05; Figure 6) at 14, 21, 28, and 42 dpi. However, antibody titers in the rOMP and FKC groups did not display any significant changes except at 42 dpi (**p < 0.01). Within the period of 14–28 dpi, the log 2 titers of the eels in two immunized groups reached above 6.0, which was higher than those of the PBS group. Serum titers in the OMP group showed an increase in the period between 14 and 42 dpi, while in the FKC group, they showed a decrease in the period from 28 to 42 dpi. The difference was not prominent in the PBS group, although it increased during the period between 28 and 42 dpi.

### 3.6 Lysozyme activity of serum, skin mucus, liver, and kidney

**Figure 7:**
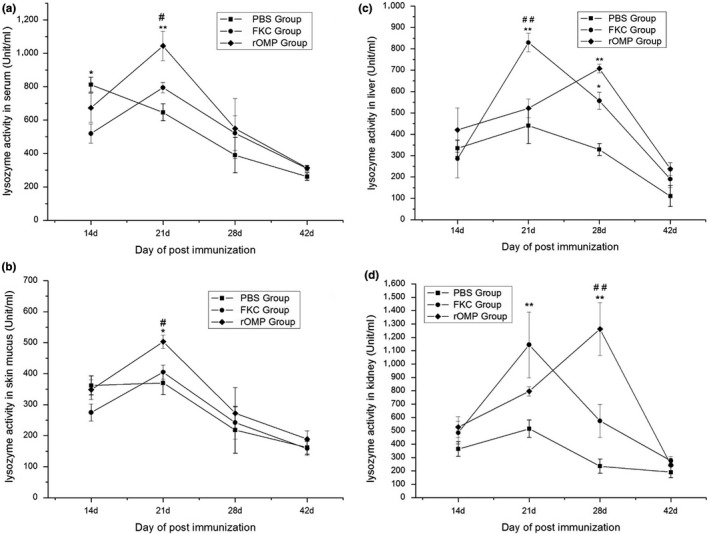
Lysozyme activity measured in serum (a), skin mucus (b), liver (c), and kidney (d) in the different immunization antigens. Data (unit/ml) are presented as means ± *SE* (*n* = 5). Significant differences were found between groups of immunized fish and group of PBS injected fish at **p < 0.01 and *p < 0.05, and between two treating groups at ^##^ *p* < 0.01 and ^#^ *p* < 0.05

Figure 7 shows the values of lysozyme activity found in serum, skin mucus, and liver and kidney lysates of vaccinated eels during the period of experimentation. The lysozyme activities in the serum of eels in the FKC group were observed to be significantly lower (*p < 0.05) than those of the control group at 14 dpi. Lysozyme activity in serum showed a definite increase (*p < 0.05) upon treatment with rOMP when compared to the other two groups at 21 dpi. At 28 and 42 dpi, lysozyme activities in the three groups exhibited a similar decrease (Figure 7a). Skin mucus from the three groups showed the same level of lysozyme activity between 14 and 42 dpi, except for the considerable difference(*p* < 0.05) between the OMP group and the other two groups at 21 dpi (Figure 7b). The lysozyme activities in the liver of the FKC group were higher (*p* < 0.01) than other groups at 21 dpi. There were also considerable differences between the OMP and control group (*p* < 0.01) and between the FKC and control group (*p* < 0.05) at 28 dpi (Figure 7c). In terms of the lysozyme activities in the kidney, the FKC group (*p* < 0.01) and OMP group showed a considerable growth at 14 and 28 dpi, respectively, compared with the control group, while important differences (*p* < 0.01) were observed between the two immunized groups at 28 dpi(Figure 7d).

## 4 DISCUSSION

It was noted that the OMPs of Gram‐negative pathogenic bacteria play an important role in bacterial interaction with the hosts and in bacterial virulence during attachment and parasitism (Liu et al., 2012; Mathur & Waldor, 2004). The pathogens have protective antigenicity to combat the host's immunological defense system, which easily recognizes the components of the OMPs as a foreign substance (Seltmann & Holst, 2013). It has been confirmed that OmpU of *V. vulnificus* elicited protective antibodies in a murine model (Duan, 2016; Jung, Park, & Heo, 2005) and the immunogenicity of the isolated OmpU of *V. vulnificus* has been proven by Western blotting (Li et al., 2013). In Japanese eel, the study of Duan (2016) showed that the relative percent survival (RPS) of OmpU versus control group after challenged by *V. vulnificus* on 28 dpi was 80%; however when challenged by *E. anguillarum*, hardly no cross‐protective effect was observed. OmpA, an OMP of *E. tarda,* also had an effective immunogenicity and protective abilities in immunized fish (Kawai et al., 2004; Duan, 2016; Kumar et al. 2007). The study of Duan (2016) also showed that the relative percent survival (RPS) of OmpA versus control group after challenged by *E. anguillarum* on 28 dpi was 77.7% in Japanese eels; however, when challenged by *V. vulnificus*, hardly no cross‐protective effect was observed.

The genus *Edwardsiella* was composed by three species *E. tarda*,* E. hoshinae,* and *E. ictaluri* before 2013 (Abayneh, Colquhoun, & Sørum, 2012). Some strains of *E. piscicida* pathogenic to fish, previously identified as *E. tarda*, was described in 2013 (Abayneh, Colquhoun, & Sørum, 2013), and a new species named *E. anguillarum*, also previously identified as *E. tarda*, was isolated from diseased eel (Reichley et al, 2017). Until 2016, 13 strains of *E. anguillarum* isolated from diseased eels in our study were first identified as *E. tarda* (Duan, 2016; Hu, 2014), and then all 13 strains were identified as *E. anguillarum* according to the method of Buján et al. (2018).

Purification of a native OMP by routine methods is a lengthy procedure associated with contamination (Su, Wan, Mohamed, & Nathan, 2010). Expression of different OMPs, such as OmpU, OmpA, OmpⅡ, and OmpTS, may serve as protective antigens against specific bacterial pathogens(Dabo, Confer, Montelongo, York, & Wyckoff, 2008; Guan et al., 2011; Khushiramani, Girisha, Karunasagar, & Karunasagar, 2007; Kumar et al., 2007), but the advantage of a combined OMP includes protection against two distinct pathogens as well as reducing the workload of heterologous expression and purification. Generally, the two genes were not full‐length, but included only the DNA sequences encoding the outer membrane domain. The DNA sequences encoding the transmembrane domain and the transmembrane signal region were removed, as these domains cannot be exposed to immune system in eels (Hu, 2014). In this study, the expression vector pGex‐2T‐*OmpU‐OmpA*‐His, which expresses a new OMP consisting of partial OmpU‐OmpA was successfully constructed. After the expressed rOMP was purified using the Ni_2_‐NTA method, it was renatured (urea) and lyophilized. This study was successful in purifying and expressing the rOMP, containing the OMPs of *V. vulnificus* (OmpU) and *E. anguillarum* (OmpA), as well as revealing the molecular masses (77.6 kD) of this rOMP using SDS‐PAGE (Figure 2). While there are some studies on differently expressed Omps or harvested natural Omps from bacterial pathogens found to have good immunogenicity (Kumar et al., 2007; Yan, Shan, Chen, & Xie, 2012), there are hardly any studies on the vaccination properties of an expressed rOMP from the pathogens *V. vulnificus* and *E. anguillarum.* Hence, this study contributes to the analysis of the immunity generated by the fusion rOMP.

It was found that the antibody response and lysozyme activity were stimulated during the defense induced by vaccination with rOMP. The results were similar in the case of *E. tarda* and *V. vulnificus* despite being immunized separately with differently expressed individual OMPs (Kawai et al., 2004; Kumar et al., 2007; Tang et al., 2010; Tian et al., 2010; Yan et al., 2012). OmpU was found to be one of the most important porins in *V. cholerae* and had also been proved to be an effective protective antigen for vaccine development (He et al., 2018; Mathur & Waldor, 2004; Wibbenmeyer, Provenzano, Landry, Klose, & Delcour, 2002). In the case of OmpA in *E. tarda*, vaccination of rainbow trout was also shown to be a good antigen (Maiti, Shetty, Shekar, Karunasagar, & Karunasagar, 2011).

Compared with the expression and purification of a single OMP in the above‐mentioned studies, the expression and purification of two OMPs required more manpower and material resources. As an alternative to the purification and expression of two individual OMPs, we constructed an expression vector that expressed two OMPs together. This finding provided evidence to the fact that the fusion rOMP in our study did not bring change in the immunogenic potency and that it stimulated a good immune response.

After infection by *V. vulnificus,* one eel from FKC group, and five eels from PBS group died, while the infection of *E. anguillarum* resulted in 3, 6, and 1 eel from FKC, PBS, and OMP groups, respectively (Figure 4). Pathogens were isolated from the injection site and the lesions in the liver and kidney. Previous studies found the RPS rates of the challenge of *A. hydrophila* and *E. anguillarum* to be 50% and 37.5%, respectively, in American eels 28 day post the immunization by a rOMP from OmpII of *A. hydrophila* and OmpS2 of *E. anguillarum* (Guo et al., 2013). In the case of infection by *A. hydrophila* or *V. vulnificus,* there was a 50% RPS after immunization by a rOMP from either *A. hydrophila* or *V. vulnificus* in American eels (Guo et al., 2015). Vaccination with rOMP resulted in 100% and 83% RPS of Japanese eels in the case of *V. vulnificus* and *E. anguillarum*, respectively. Except for the different eels, an equal concentration of incomplete Freund's adjuvant was administered to the three groups of fish in this study, but no adjuvant was added in the PBS and FKC groups in the studies referred to in Guo et al. (2015, 2013 ). However, it is important to note that the dose of pathogens was five times greater in those two previous studies than it was in this study. Therefore, it can be presumed that a lower dose of pathogens results in a high RPS rate.

Proliferation of eel whole blood cells resulted with a little wide range of stimulation index (SI), and it had previously been reported in European eels (Guo, Yang, Feng, Duan, & Zhao, 2016). ConA is a T lymphocyte stimulator, and lymphocytes should usually be isolated for this study, but it had been found that ConA specifically stimulate T lymphocyte proliferation in the culture of whole blood cells. Our previous studies found that some of the eel's lymphocytes were difficult to isolate, and the proliferation of lymphocytes could be measured by whole blood culture (Guo & Guan, 2009). At 14 day post vaccination, proliferation of whole blood cells in the two experimental groups was higher than that of the PBS group, but the SI in the eels of the FKC group was significantly higher (*p* < 0.01) than that of the other two groups 28 day post immunization (Figure 5). Other studies on the immunogenicity of the OMP showed that the proliferation of whole blood cells in the OMP group was significantly higher than that in the PBS control group, probably because of the absence of Freund's adjuvant in the control group (Guo et al., 2015, 2013 ). As the incomplete Freund's adjuvant usually improves cellular immunity in animals; the different results between our and previous studies are probably due to the inclusion of incomplete Freund's adjuvant.

In this study, significant antibody titers were detected in eels of OMP and FKC groups with a maximum at 28 and 42 day post vaccination(Figure 6). The results were optimal for eels in the OMP group during the experimental period, and antibody titers in this group seemed to be increased by 42 day. Additionally, antibody levels in the FKC group decreased and were significantly lower than those of the OMP group at 42 day, indicating that the immunogenicity of OMP was probably better than FKC (Guo et al., 2015; Khushiramani et al., 2007). It was worth mentioning that the RPS rates were in accordance with the antibody titers at 28 day post immunization, indicating the level of antibody titers was positively correlated with the protective effect in the eels in the FKC and rOMP groups. Standard errors pertaining to the titers of the control antibody in the PBS group between 14 and 42 days varied, perhaps resulting from the different breeds among the eel population used in this study. This phenomenon is common in the American eel (Guo et al., 2015) and the large yellow croaker (Mao et al., 2007). In our previous study, antibody titers were also significant at 14 day post immunization between the PBS and rOMP groups of *A. hydrophila* and *E. anguillarum* and between the PBS and rOMP groups of *A. hydrophila* and *V. vulnificus* (Guo et al., 2015, 2013 ). The difference was probably due to the different rOMP constructed between the previous (OmpS2 of *E. anguillarum*) and the present study (OmpA of *E. anguillarum*). Interestingly, when coating rOMP as antigen in the ELISA assay, hardly no anti‐rOMP antibody was detected in the serum of eels immunized FKC, but when coating boiled suspension of *E. anguillarum* and *V. vulnificus*, titers of anti‐*E. anguillarum* and *V. vulnificus* antibody in the OMP group was the same as coating the rOMP (data not shown in this study). Reasons of no anti‐rOMP antibody in eels immunized FKC probably due to the competition of complex antigens in the bacterial cells.

There are differences between the nonspecific phagocytosis of granulocytes and the specific phagocytosis mediated by antibodies. Lysozyme activity may differ between the control and the FKC groups since the bacteria ghosts also stimulate the release of lysozyme through granulocytes, but the stimulation is possible weakened by the presence of adjuvants. OMP itself can not stimulate the production of lysozyme in granulocytes, but it is worth to detect whether it enhances the stimulation of adjuvant to granulocytes when the OMP is fully mixed with the adjuvant. Lysozyme activity in this study was found to be higher in the two vaccinated groups than in the control group (Figure 7). The highest lysozyme activity was observed in the samples prepared from kidney, followed by the serum, liver, and skin mucus, indicating that the role of kidney is crucial in the nonspecific immunity (Kawahara & Kusuda, 1988). The increase of lysozyme activity was observed in the two treated groups at 21 and 28 days with respect to the four tissues, and decreased at 42 days (Figure 5), demonstrating the possible restriction stimulated by vaccination in the later period. Interestingly, the RPS rates also corresponded with lysozyme activity in the liver and kidney at 28 day post immunization. The high lysozyme activity in the liver and kidney at 28 day post immunization would enhance the antibacterial capacity, providing good protective effects in eels in the FKC and rOMP groups. At 42 days, the three groups showed the same level of lysozyme activity, indicating the significant role of rOMP and FKC at the early stages in the production of lysozyme in eels. Lysozyme activity in the skin mucus stayed constant in the three groups except on 21 days, which was in accordance with previous studies (Guo et al., 2015, 2013 ). Significant differences in lysozyme activities between the two treated groups in eels could probably be attributed both to the immunized antigens and Freund's adjuvant (Ackerman, Iwama, & Thornton, 2000; Esteve‐Gassent et al., 2004).

## 5 CONCLUDING REMARKS

Our results confirmed the immunization with the recombinant fusion protein (OmpU of *V. vulnificus* and OmpA of *E. anguillarum*), with the aid of a singular antigen stimulates a defensive mechanism of resistance to the two pathogens in Japanese eels (*A. japonica*). The immunity was the result of higher levels of specific antibodies in serum and lysozyme activity in the kidney and liver. To develop effective vaccines against *V. vulnificus* and *E. anguillarum*, immunogenic OMPs from these bacterial pathogens should be given more attention, especially on the recombinant protective antigens of fusion OMPs of two or more pathogens. In this study, the rOMP is more effective than the bivalent FKC vaccine. However, the efficacy of the FKC vaccine would be limited if the serotype of the pathogen changed (Mao, Lou, Wu, & Nan, 2003). Since different serotypes of the pathogens generally have the same OMP gene sequence, protective subunit vaccines generated from the fusion of two or more OMPs are in need of further study to ensure both the efficacy and economy of vaccination in aquaculture.

## CONFLICT OF INTEREST

The authors declare no financial or commercial conflict of interest.

## AUTHORS CONTRIBUTION

Guo and Yan conceived the project, contributed to analysis and interpretation of data and participated in draft preparation and wrote the manuscript. Guo, Hu, Feng, Lin and He performed experiments. Guo and Hu prepared the figures and wrote materials and methods section. All authors discussed and proofread the work and manuscript.

## ETHICS STATEMENT

We state that eels used in our study were cultured under protocols approved by the Jimei University Animal Care Committee (2011–59) and all experiments complied with the current laws of China and international organizations for the use of experimental animals.

## Data Availability

All data associated with the article have been included in this manuscript.
